# An ACE2-Based Decoy Inhibitor Effectively Neutralizes SARS-CoV-2 Omicron BA.5 Variant

**DOI:** 10.3390/v14112387

**Published:** 2022-10-28

**Authors:** Haoran Zhang, Bing Hu, Panjing Lv, Yahui Liu, Meng Guo, Zhi Wu, Kangping Zhou, Minglu Dai, Xiao Yu, Zhang Liu, Bo Yu, Liqiong Xu, Min Guo, Kun Cai, Yan Li

**Affiliations:** 1 Department of Pathogen Biology, School of Basic Medicine, Tongji Medical College, Huazhong University of Science and Technology, 13 Hangkong Road, Wuhan 430030, China; 2 Institute of Health Inspection and Testing, Hubei Provincial Center for Disease Control and Prevention (Hubei CDC), Wuhan 430079, China; 3 Kangma Healthcode (Shanghai) Biotech, Co., Ltd., 118 Furonghua Road, Building No. 15, Shanghai 201203, China; 4 Department of Pediatrics, Tongji Hospital, Tongji Medical College, Huazhong University of Science and Technology, Wuhan 430030, China

**Keywords:** SARS-CoV-2, Omicron BA.5, protein antagonist, ACE2, neutralization escape

## Abstract

The recently circulating SARS-CoV-2 Omicron BA.5 is rampaging the world with elevated transmissibility compared to the original SARS-CoV-2 strain. Immune escape of BA.5 was observed after treatment with many monoclonal antibodies, calling for broad-spectrum, immune-escape-evading therapeutics. In retrospect, we previously reported Kansetin as an ACE2 mimetic and a protein antagonist against SARS-CoV-2, which proved potent neutralization bioactivity on the Reference, Alpha, Beta, Delta, and Omicron strains of SARS-CoV-2. Since BA.5 is expected to rely on the interaction of the Spike complex with human ACE2 for cell entry, we reasonably assumed the lasting efficacy of the ACE2-mimicking Kansetin for neutralizing the new SARS-CoV-2 variant. The investigation was accordingly performed on in vitro Kansetin-Spike binding affinity by SPR and cell infection inhibition ability with pseudovirus and live virus assays. As a result, Kansetin showed dissociation constant K_D_ and half inhibition concentration IC_50_ at the nanomolar to picomolar level, featuring a competent inhibition effect against the BA.5 sublineage. Conclusively, Kansetin is expected to be a promising therapeutic option against BA.5 and future SARS-CoV-2 sublineages.

## 1. Introduction

The novel coronavirus disease 2019 (COVID-19) has resulted in over 600 million cumulative cases and over 6 million cumulative deaths worldwide (as of September 2022) [1]. It is caused by the infection of severe acute respiratory syndrome coronavirus 2 (SARS-CoV-2) [2]. SARS-CoV-2 is a positive-sense single-stranded RNA virus that belongs to the Beta-CoVs genus of the *Coronaviridae* family [3] and is a zoonotic pathogen that can cause severe respiratory diseases in humans [4]. Mutations in the SARS-CoV-2 genome occur spontaneously and randomly [5], with most having no apparent effect on the virus. However, a few critical mutations can affect virulence, transmissibility, and severity of clinical manifestations of the virus, leading to the emergence of variants of concern (VOCs) and conferring the virus to potentially evade pre-existing antiviral drugs and neutralizing antibodies [6,7]. Therefore, there is an urgent need to design antiviral drugs to curb this deadly and variable virus.

Previously, we designed a protein antagonist Kansetin that confers neutralization against SARS-CoV-2 and several significant VOCs, including Omicron BA.2 [8]. However, the recently emerging SARS-CoV-2 variant Omicron BA.5, a VOC first detected and reported in South Africa, is now spreading worldwide at a rapid pace [9,10]. While a sublineage of the Omicron BA.2 lineage, BA.5 has replaced BA.2 as one of the highest circulating VOCs [11]. The overwhelming transmissibility of BA.5 was speculated to be connected to its immune escape capability against SARS-CoV-2 vaccines and monoclonal antibodies (mAbs) [12]. The BA.5 variant has been reported to show broad resistance to antibody-involved neutralization, possibly attributed to decisive mutations in receptor binding domain (RBD) epitopes of the SARS-CoV-2 Spike protein (S protein) [13]. In recent pseudovirus neutralization and live virus focus reduction neutralization tests, the mAbs that used to be effective on earlier SARS-CoV-2 strains such as casirivimab, bamlanivimab, etesevimab, and sotrovimab displayed a severe reduction in inhibitory efficacy against BA.5 [12,14,15]. Hence, alternative therapeutic options, such as antivirals and human ACE2 (hACE2) mimetics, may end up under favorable consideration to counteract the BA.5-oriented COVID-19 pandemic. Here, we report the inspiring BA.5 neutralization potency of Kansetin, a novelly designed hACE2-mimicking protein antagonist.

## 2. Materials and Methods

### 2.1. Cell-Free Synthesis of Kansetin

We adapted the Spike-binding domain (SBD) (19–394 a.a.) of human ACE2 (UniProt ID: Q9BYF1), which predominates in the direct interaction with Spike protein of SARS-CoV-2, into Kansetin as the antagonistic functional domains against SARS-CoV-2. The plasmid of Kansetin was constructed on the pD2P vector and amplified by the Ampi system. The amplified plasmid was added to the cell-free protein synthesis system (Kangma Healthcode, Shanghai, China) at a volume ratio of 1:30 and incubated at 30 °C for 4 h for the expression of Kansetin. The reaction mixtures were centrifuged at 4000 rpm for 10 min, and the supernatant was collected for subsequent purification. The magnetic His Monster Beads (Kangma Healthcode, Shanghai, China) were incubated with harvested supernatant for 1 h with rotation at 4 °C, washed three times with wash buffer (50 mM Tris-HCl, pH 8.0, 500 mM NaCl, 10 mM imidazole), and finally eluted target protein with elution buffer (50 mM Tris-HCl, pH 8.0, 500 mM NaCl, 250 mM imidazole) [8]. To avoid interference of high concentrations of imidazole with subsequent cellular experiments, the eluted target proteins were ultrafiltered to remove excess imidazole.

### 2.2. Surface Plasmon Resonance (SPR) Experiment

SPR studies were performed on an OpenSPRTM device (Nicoya Lifesciences, Kitchener, Canada). The assays were performed at 25 °C with running buffer (10 mM HEPES, pH 7.4, 150 mM NaCl, 3 mM EDTA, and 0.05% Tween-20). The Sensor Chip COOH (Nicoya Lifesciences, Kitchener, Canada) was equilibrated in running buffer before the covalent coupling of purified Kansetin. The His-tagged RBD of the SARS-CoV-2 Omicron BA.5 strain (Sino Biological Inc, Beijing, China) was diluted to a two-fold dilution series (0, 10, 20, 40, 80, and 160 nM), and the Spike S1+S2 trimer of the BA.5 strain (Sino Biological Inc, Beijing, China) was diluted to a two-fold dilution series (0, 2.5, 5, 10, and 20 nM). Each sample was run across the chip with running buffer for association for 240 s and with HEPES-ET + running buffer for dissociation for 360 s at a flow rate of 20 μL/min, another blank channel set as a negative control. The dissociation constant K_D_ was calculated using the TraceDrawer software (Ridgeview Instruments ab, Uppsala, Sweden).

### 2.3. Pseudovirus Neutralization Assay

The 293T-ACE2 cells, pseudoviruses, and luciferase assay system were purchased from Genomeditech. This pseudovirus was generated based on the backbone of the VSVG pseudotype virus, and the lentivirus envelope protein VSVG was replaced by SARS-CoV-2 BA.5 Spike protein. Co-transfection of the backbone with lentivirus packaging plasmid and CMV-GFP-T2A-Luciferase plasmid into 293T-ACE2 cells allowed the assembly of pseudovirus with infectious cell activity and without autonomous replication ability.

The 293T-ACE2 cells (1.5 × 10^4^ cells/well) were seeded into 48-well plates. After incubation at room temperature for 1 h, the mixture containing 100 µL of Kansetin and 100 µL of pseudovirus was added to the 48-well 293T-ACE2 cells to detect pseudoviral infectivity. The culture medium was refreshed at 6 h after infection, and the expression of eGFP in infected cells was determined by fluorescence microscopy at 48 h post-infection. Six replicate wells were made for each concentration of Kansetin. The buffer was used as a blank control for the experiment and showed no inhibitory effect on pseudovirus-infected cells. The data were processed using Prism software (GraphPad Prism 9.0).

### 2.4. Live Virus Neutralization Assay

The SARS-CoV-2 Omicron BA.5 variant (YJ20220701-01) was provided by Hubei Provincial Centre for Disease Control and Prevention. The SARS-CoV-2 live virus experiments were performed in the Biosafety level 3 (BSL-3) facility at the Institute of Health Inspection and Testing, Hubei Provincial Center for Disease Control and Prevention. The Vero E6 cells were provided by Hubei Provincial Centre for Disease Control and Prevention.

SARS-CoV-2 BA.5 live viruses at 100 TCID_50_ were prediluted in DMEM with 2% fetal bovine serum (FBS). Eleven two-fold dilutions (starting from a 1:80 sample dilution) of Kansetin were mixed with live viruses at 100 TCID_50_ in equal volume and incubated for 1 h at 37 °C. Antagonist-virus complexes were added to Vero E6 monolayers in 96-well plates and cultured for 72 h at 37 °C, and observed the cytopathic effect (CPE) via a microscope. Four replicate wells were set up for each sample, and the buffer was used as a blank control in each experiment. The percentage of wells without cytopathic effect was observed and calculated as the percentage of inhibition. Live virus neutralization assay data were processed using Prism software (GraphPad Prism 9.0).

## 3. Results and Discussion

Kansetin is a protein homooligomer, with each monomeric unit containing two S protein binding domains (SBD) extracted from the hACE2 binding interface of S protein RBD [8]. This tightly packed complex was observed to cluster multiple SARS-CoV-2 viral particles into bulky aggregates. Kansetin was tested against SARS-CoV-2 Wuhan-hu-1 strain (reference strain) and several VOCs, including Delta and Omicron BA.1 strain. In vitro surface plasmon resonance (SPR) assay recorded Kansetin-RBD binding affinity at a nanomolar level, and pseudovirus test and live virus test likewise returned picomolar IC_50_ values at best. Kansetin’s broad-spectrum VOC neutralization capability was expected to result from the evolutionarily stable hACE2 binding ability of S protein, given the significance of hACE2 in SARS-CoV-2′s cell entry [16]. Thus, we assume Kansetin to be, at least to a certain extent, effective in neutralizing BA.5 as this new subvariant exhibited tight binding with hACE2 (K_D_ = 25 nM) similar to BA.1 [17].

**Figure 1:**
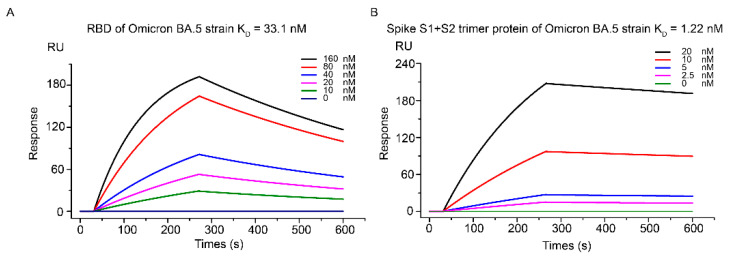
SPR-based characterization of Kansetin’s binding affinity to Spike RBD protein of SARS-CoV-2 Omicron BA.5 strain (**A**) and Spike S1+S2 trimer protein (**B**).

Accordingly, we first analyzed Kansetin’s binding activity to both BA.5 S1+S2 protein trimer and standalone RBD via the SPR assay. The K_D_ was determined to be 1.22 nM and 33.1 nM, respectively (Figure 1A,B). Comparable to the previous SPR data from Kansetin and earlier VOCs (Table 1), the nanomolar Kansetin-BA.5 affinity suggested strong competitive binding to BA.5 S protein regarding the hACE2 native to host cells. 

**Figure 2:**
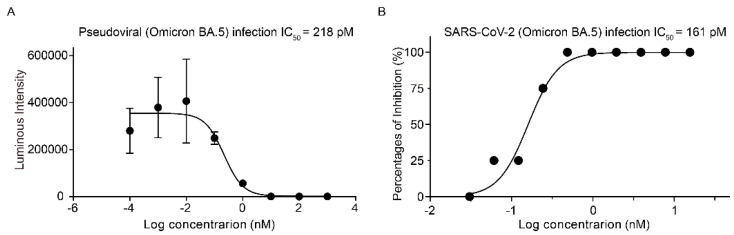
The inhibitory effect of Kansetin on Omicron BA.5 strain. (**A**) Kansetin neutralizes pseudovirus infection of 293T-ACE2 cells. Data are mean ± SD (*n* = 6 per group). The buffer was used as a blank control for the experiment and showed no inhibitory effect on pseudovirus-infected cells. (**B**) Kansetin neutralizes live virus infection of Vero E6 cells. The buffer was used as a blank control in each experiment, which has no inhibitory effect on live virus-infected cells.

For further validation of the SPR results, we characterized Kansetin’s cell infection inhibition capability via SARS-CoV-2 BA.5 pseudovirus neutralization experiment. We observed no significant evasion of Kansetin for the BA.5 variant (IC_50_ = 218 pM), a readout consistent with the SPR assay (Figure 2A). Subsequently, a live virus neutralization test was performed for validation, yielding an IC_50_ value of 161 pM (Figure 2B). Notably, these results exhibited a slightly decreased but still potent BA.5 neutralization ability of Kansetin compared to the reference and other VOCs (Table 1) [8], reaffirming Kansetin as a competent inhibitor of SARS-CoV-2 BA.5 infection.

**Figure 3:**
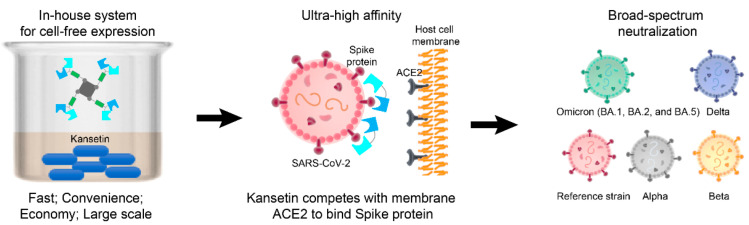
Schematic depiction of expression and strong SARS-CoV-2 neutralization of Kansetin.

Discussion of why Kansetin maintained its neutralization ability when many antibodies did not may help expand the inhibitor’s potential. Notably, in the BA.5 RBD sequence, the L452R substitution was disclosed to engender more severe immune escape compared to the moderate mutations such as L452M in BA.2.13 or L452Q in BA.2.12.1, while the F486V mutation reduces the hACE2 binding affinity via decreased hydrophobic interaction [18]. Several hACE2-competing antibodies possess an epitope encompassing site L452 and thus are highly sensitive to the L452R mutation in BA.5, contributing to the immune escape [18]. Conversely, the hACE2-RBD interaction in the reference or BA.2 strain follows a different pattern where the L452 was not in direct contact with hACE2 [19,20]. Consequently, the L452R substitution featured in BA.5 might not be expected to influence its hACE2 binding ability as F486V does. In retrospect, Kansetin also exhibited analogous neutralization to earlier VOCs, including Alpha, Beta, Delta, and Omicron BA.1 strains, showing its sustainable RBD-targeting competence in the long term (Figure 3) [8]. These combined factors might contribute to understanding the still strong BA.5 binding affinity and neutralization ability of Kansetin, which in nature is a hACE2 mimetic with efficacy-enhancing modifications.

Another distinguishing aspect of Kansetin as a SARS-CoV-2 antagonist is its storage- and user-friendly properties. mAbs are known to suffer from loss of bioactivity due to frozen-state storage [21], a factor unfavorable for extensive therapeutic application. Furthermore, mAbs might cause serious adverse effects such as thyroid diseases, dermatitis, and cardiotoxicity [22], a safety problem that needs extra attention for therapists. These properties of concern were one of the driving forces that helped us develop Kansetin by rational design. Our previous characterization of toxicity and storability identified Kansetin as an ultra-stable protein at room temperature with no observable toxicity [8]. Notably, Kansetin possesses specific properties that distinguish the antagonist from mAbs in a potentially disadvantageous way. Although accurate immunogenicity prediction is challenging due to many complex causation factors, mAbs-bearing human sequences are expected to display less immunogenicity in general [23]. For Kansetin, potential immunogenic problems might arise due to artificial arms other than hACE2-extracted SBDs. Another concern is that the α1-α2 helices of hACE2 are the component of a cavity for Angiotensin II binding and catalysis [24]. Therefore, the reaction of human Angiotensin II pathways to the intake of Kansetin and its possible consequences needs extra care taken. Nevertheless, combined with the promising potency and patient-friendly properties, planned forthcoming clinical trials are expected to further expand the landscape of Kansetin’s practical applications.

In summary, we reported the retained neutralization efficacy of SARS-CoV-2 inhibitor Kansetin against the currently circulating Omicron BA.5 subvariant by in vitro binding affinity assays, pseudovirus neutralization, and live virus neutralization assays. Historical behaviors of the COVID-19 pandemic, such as its high mutation rate and increased transmission capacity, serve as a reminder that medical solutions should be provided as efficiently and practically as possible. Our results responded with a potential therapeutic option for preventing and treating COVID-19 with the predominance of BA.5 and, if possible, other variants in the future as long as the ACE2 binding is still a vital evolutionary standard for SARS-CoV-2.

## Figures and Tables

**Table 1:** Summarization of data between different SARS-CoV-2 strains ^1^.

Strains	Analytes	K_D_ (nM)	IC_50_ (pM) ^2^
Reference	RBD	1.25	108.6
Alpha	RBD	-	92.8
Beta	RBD	-	121.9
Delta	RBD	0.837	61
Omicron BA.1	RBD	0.656	121.9
Omicron BA.2	RBD	-	134
Omicron BA.5	RBD	33.1	161
	S1 + S2 trimer	1.22	

^1^ This table summarizes the data for BA.5 in this study and other data quoted from our previous work [8]. ^2^ The IC_50_ data refer to those determined by live virus neutralization assay.

## Data Availability

The datasets used and/or analyzed during the current study are available from the corresponding author upon reasonable request.
